# Differential expression of long non-coding RNA in the hypothalamus-pituitary-gonadal axis of Wanxi white geese during laying and broodiness periods

**DOI:** 10.5713/ab.25.0348

**Published:** 2025-10-22

**Authors:** Xiaojin Li, Mengmeng Hou, Yuhua Wang, Fou Wu, Xinwei Tong, Fei Xie, Changsheng Jiang, Mengmeng Jin, Man Ren, Shenghe Li

**Affiliations:** 1 College of Animal Science, Anhui Science and Technology University, Chuzhou, China; 2 Anhui Province Key Laboratory of Animal Nutritional Regulation and Health, Chuzhou, China; 3 Local Geese Gene Bank in Anhui Province, Chuzhou, China

**Keywords:** Broodiness Behavior, Hypothalamic-Pituitary-Gonadal Axis, Laying Performance, Long Non-coding RNA, Wanxi White Goose

## Abstract

**Objective:**

This study explored the molecular mechanism of the hypothalamus-pituitary-gonadal (HPG) axis on the regulation of brooding behavior and laying performance of Wanxi white geese (WWG). The transcriptome of the hypothalamus, pituitary, and ovarian tissues of laying and brooding WWG was sequenced to identify genes and long non-coding RNAs (lncRNAs) that may be important in regulating the egg-laying performance and broodiness behavior of WWG.

**Methods:**

This study sequenced the lncRNA on the hypothalamus, pituitary, and ovarian tissues of WWG white geese during laying and broodiness periods to determine the differentially expressed lncRNA (DElncRNA) in the hypothalamus-pituitary-ovary axis. lncRNA-microRNA (miRNA)-messenger RNA (mRNA) (ceRNA) regulatory network was constructed using selected differentially expressed genes (DEGs), differentially expressed microRNAs (DEMs), and DElncRNAs. Differentially expressed DEGs, DEMs, and DElncRNAs were further confirmed via real-time quantitative polymerase chain reaction. The dual luciferase reporter gene assay confirmed a targeting relationship between the MSTRG.1166. 1/miR-450-x/SOX8 axis.

**Results:**

A total of 184 (brooding period hypothalamus vs laying period hypothalamus), 180 (brooding period pituitary vs laying period pituitary), and 880 (brooding period ovary vs laying period ovary) DElncRNAs were screened. Gene Ontology and Kyoto Encyclopedia of Genes and Genomes functional enrichment analysis showed that the DElncRNAs significantly enriched Steroid hormone biosynthesis, Neuroactive ligand-receptor interaction, Calcium signaling, and other pathways. The ceRNA regulatory network of laying performance and nesting behavior was constructed through the database. MSTRG.1166.1-miR-450-x-*SOX8*, MSTRG. 7163.5-miR-182-x-*CSMD1*, XR_007167835.1-miR-277-z-*RAB3B*, MSTRG. 7163.5-miR-151-y-*PAQR9*, MSTRG.4615.2-miR-96-x-*DAPK1*, XR_ 007164924.1-miR-144-*y-TFPI*, XR_007161186.1-miR-205-x-*THRB*, MSTRG.10196.1/ XR_001206277.2-miR-339-*x-TRAF4*, and MSTRG.9442.1-miR-9-y-*FBN3* may play an important role in the ovarian development of WWG. The dual luciferase reporter gene assay confirmed a targeting relationship between the MSTRG.1166.1/miR-450-x/*SOX8* axis. The results of this study systematically expounded on how the HPG axis involves lncRNA, miRNA, and mRNA to post-transcriptionally regulate the broodiness behavior and laying performance of WWG.

**Conclusion:**

The results will improve knowledge of the complex interaction between lncRNA and genes controlling laying performance and broodiness behavior.

## INTRODUCTION

Wanxi white geese (WWG) is an excellent geese breed in China, characterized by strong disease resistance, good meat quality, and geese down quality. However, long-term natural hatching causes reproductive cycle segmentation, forming a strong broodiness in WWG [1]. Brooding, an instinct of hens after laying eggs, is an important part of complete reproductive behavior that involves the hypothalamus-pituitary-gonad (HPG) axis, a system composed of endocrine glands.

During broodiness, the level of prolactin (PRL) secreted by anterior pituitary increases, inhibiting gonadotropin-releasing hormone (GnRH) secretion by the hypothalamus, thereby reducing follicle-stimulating hormone (FSH) and luteinizing hormone (LH) secretion by the pituitary [2], leading to follicular development to stop and ovarian atrophy. At the laying period, the ovary secretes progesterone and estrogen, produces oocytes, and promotes ovulation. The HPG axis influences and regulates follicular development, maturation, and ovulation, affecting the laying cycle and reproductive efficiency. The hypothalamus secretes GnRH, which stimulates the anterior pituitary gland to secrete gonadotropins, LH, and FSH. Moreover, the hypothalamus acts on the ovary to secrete estrogen, regulate ovulation, and ultimately regulate egg production.

Ovarian follicular development determines egg-laying performance and broodiness in geese. Thus, determining the factors affecting ovarian follicular development has been the focus of recent research. Researchers have been searching for key candidate genes involved in follicular development. However, most of the work has focused on protein-coding genes. However, more evidence has shown that long non-coding RNA (lncRNA) is an important regulator of cell function in different tissues [3]. lncRNAs are non-coding RNAs with >200 nucleotides and mainly affect gene expression through cis (cis) and trans (trans) regulatory mechanisms. Some lncRNAs play an important biological role in poultry ovaries and affect the reproductive performance of poultry. Wu et al [4] found that lncRNAs participate in the lncRNA-microRNA (miRNA)-messenger RNA (mRNA) (ceRNA) co-expression network, potentially affecting the development of duck follicles. Huang et al [5] found that lncRNAs can regulate hormone sensitivity in the ovary of laying hens. Furthermore, lncRNAs affect follicular development and ovulation through FSH and LH signaling pathways. Another study analyzed the transcriptome (RNA-seq) of the hypothalamus and pituitary to compare the laying performance of high-yield and low-yield laying Chinese Big Bone Chicken (CDC) [6]. Nonetheless, most present studies focus on screening mRNAs and miRNAs that regulate broodiness behavior without analyzing the regulation of ceRNA interaction network on laying performance and broodiness behavior of geese.

Thus, this study sequenced the lncRNA on the hypothalamus, pituitary, and ovarian tissues of WWG white geese during laying and broodiness periods to determine the differentially expressed lncRNA (DElncRNA) in the hypothalamus-pituitary-ovary axis. The ceRNA network revealed the effect of lncRNA on the HPG axis development and egg production performance. The results provide a new perspective for further studies on the molecular mechanism of HPG axis development in WWG and a theoretical basis for improving its reproductive performance.

## MATERIALS AND METHODS

### Sample collection

The geese used in this study were obtained from Dingyuan Junming Ecological Farm and were reared under standardized feeding management practices in a controlled environment. After the geese began to lay eggs, a total of 100 geese were selected for individual cage rearing. Among them, 10 geese that had laid more than 5 eggs were chosen as the laying geese, and 10 geese that had been brooding continuously for more than 3 days were chosen as the brooding geese. Then, the egg-laying conditions of the laying and brooding geese were observed and recorded. Based on the behaviour of WWG, the birds were divided into laying and broodiness groups, each with 10 geese and separately fed. The eggs produced by the geese during the laying and broodiness periods were observed and counted daily for 10 days. All geese were euthanised by cervical dislocation, and the hypothalamus, pituitary gland, and ovaries were immediately excised and rinsed with phosphate-buffered saline (PBS) buffer. Serum was collected for subsequent hormone levels and serum biochemical determination. The samples were snap-frozen in liquid nitrogen and stored at −80°C for total RNA extraction. Laying period ovary (LO), laying period hypothalamus (LH) and laying period pituitary (LP) were labeled LO1-3, LH1-3, and LP1-3, respectively. Brooding period ovary (BO), brooding period hypothalamus (BH), and brooding period pituitary (BP) were labeled BO1-3, BH1-3, and BP1-3, respectively. Finally, Gideo Biotechnology constructed the libraries and sequenced the samples. The specific steps were as follows: Total RNA was extracted using the Trizol kit (Invitrogen) according to the manufacturer’s instructions, and its quality was assessed with the Agilent Technologies 2100 Bioanalyzer (Agilent Technologies). The RNA was then purified and repaired using the QiaQuick polymerase chain reaction (PCR) kit (QIAGEN). Transcriptome sequencing libraries were prepared using the Illumina TruSeq kit (Illumina) and sequenced on the Illumina HiSeqTM 4000 (Illumina).

### Determination of hormone levels

Serum hormone detection adopteda competitive radioimmunoassay using Iodine, including PRL, FSH, estradiol (E2), vasoactive intestinal peptide (VIP), progesterone (P4), LH, dopamine (DA), GnRH. Kit provided by Beijing North Biological Bio-technology Research Institute), and were measured on a radioimmunoassay system (radiometer, Xi’an Nuclear Instrument Factory xh6080).

### Determination of serum biochemical indexes

A Cenece DL-5m low-speed refrigerated centrifuge was used to centrifuge the samples at 3,000 r/min for 15 min. We then separate the serum and store the separated serum samples at 20°C.Therefore, we used previously isolated sera in subsequent hormone assays. The serum biochemical measurement indices include alanine aminotransferase (ALT), alkaline phosphatase (ALP), totalprotein (TP), albumin (ALB), triglycerides (TG), globulin (GLB), glucose (GLU), total cholesterol (TCZ) and others. The measurement was conducted using theUniCelDX800 Synchron fully automated biochemical analyzer system.

### Transcriptome data analysis

The original sequences (raw reads) were subjected to fastp for quality control and filtering of low-quality reads to obtain clean reads [7]. Next, Hisat2 (v2.0.4) [8] software was used to compare the selected clean reads with the reference genome (NCBI_GCF_002166845.1) to identify and quantify the gene expression. Using Fragments Per Kilobase of exon model per Million mapped fragments (FPKM) with StringTie (v1.3.1), mRNA expression levels were evaluated. Finally, HTSeq was used to analyze gene expression, and significantly differentially expressed genes (DEGs) were identified using edgeR (p-values<0.05, |log_2_FC|≥0.26).

### Long non-coding RNA data analysis and target gene prediction

Based on the assembled transcripts, we applied structural and functional criteria specific to lncRNAs≥2 exons and a length>200 bp to filter the initial set. For all reconstructed transcripts, we integrated the current mainstream protein-coding potential assessment tools CNCI [9], CPC2 [10], CPAT [11], and PFAM [12] to perform the screening. The intersection of transcripts with no protein-coding potential identified in the analysis results was taken as the set of lncRNAs predicted by this study and that of |log_2_FC|>0.26, p*-*values*<*0.05 was considered significantly different. Protein-coding genes located within 100 kb upstream and downstream of differential lncRNAs were considered cis-target genes of differential lncRNAs. Pearson correlation was used to analyze the correlation between lncRNA and protein-coding genes between samples. Protein-coding genes whose absolute correlation values exceeded 0.95 were considered significant at p<0.05 and were used as trans-target lncRNA genes.

### Gene Ontology and Kyoto Encyclopedia of Genes and Genomes enrichment analysis of long non-coding RNA target genes

Gene Ontology (GO) and Kyoto Encyclopedia of Genes and Genomes (KEGG) enrichment analysis of lncRNA target genes was performed using ClusterProfiler and KOBAS software. The GO term and KEGG pathway with p*<*0.05 were significantly enriched, and those with p*<*0.01 were extremely significantly enriched.

### Construction of long non-coding RNA-microRNA-messenger RNA interaction network

RNAhybrid, miRanda, and TargetScan software were used to predict the relationship between differentially expressed miRNAs and target genes (lncRNAs and mRNAs). The Spearman rank correlation coefficient (SCC) between miRNA, lncRNA and mRNA was calculated for the above target genes, and the expression correlation between lncRNA and mRNA was analyzed using the Pearson correlation coefficient (PCC) [13]. Based on the ceRNA mechanism [14], we selected only those miRNAs that have established targeting interactions with both the candidate lncRNAs and their corresponding mRNAs. The expression was considered negatively correlated if (SCC≤−0.7), the expression level between candidate lncRNA and mRNA was positively correlated (PCC>0.9), and the hypergeometric distribution was significant (p<0.05) as the final ceRNA co-expression network analysis results. Cytoscape software was used to visualize the key regulatory network modules.

### Validation using real-time fluorescence quantitative reverse transcription polymerase chain reaction

Primers were designed using Oligo 7 and miRNA Design software. The primer sequences (Supplement 1) were synthesized by Shenggong Bioengineering. 4 DELncRNAs, differentially expressed microRNAs (DEGs), and DEMs were randomly selected to verify the accuracy of the sequencing results, with *GAPDH* as the internal reference of lncRNA and mRNA and U6 as the internal reference of miRNA. The mRNAs were quantified using SYBR quantitative polymerase chain reaction (qPCR) Master Mix (EZBioscience), while miRNAs were quantified using miRNA Universal miRNA SYBR qPCR Master Mix (Vazyme). The relative expression of DEGs, DEMs, and lncRNAs was calculated by the 2^−ΔΔ^CT method. GraphPad Prism 3.8 was used to analyze the data obtained.

### Dual-luciferase reporter assay system

The binding between *SOX8* mRNA 3′UTR and miR-450-x, MSTRG.1166.1, and miR-450-x was verified using the dual luciferase reporter gene assay. To do this, SOX8-WT and SOX8-MUT, MSTRG-WT, and MSTRG-MUT plasmids were constructed using the vector psiCHECK2 (Promega). HEK293T cells were cultured for 24 hours. When they reached 70%–80% density, the cells were transfected with Lip3000 for 48 hours, the firefly and renilla luciferases were added, and the shock plate was mixed to detect the firefly and renilla luciferase activities.

## RESULTS

### Long non-coding RNA genome characteristics

The transcript length, exon number, ORF length, and expression levels of 9,514 known lncRNAs and 4,541 novel lncRNAs were counted and compared with mRNA to reveal the lncRNA genomic characteristics in the ovary, pituitary, and hypothalamus of the WWG.

**Figure 1:**
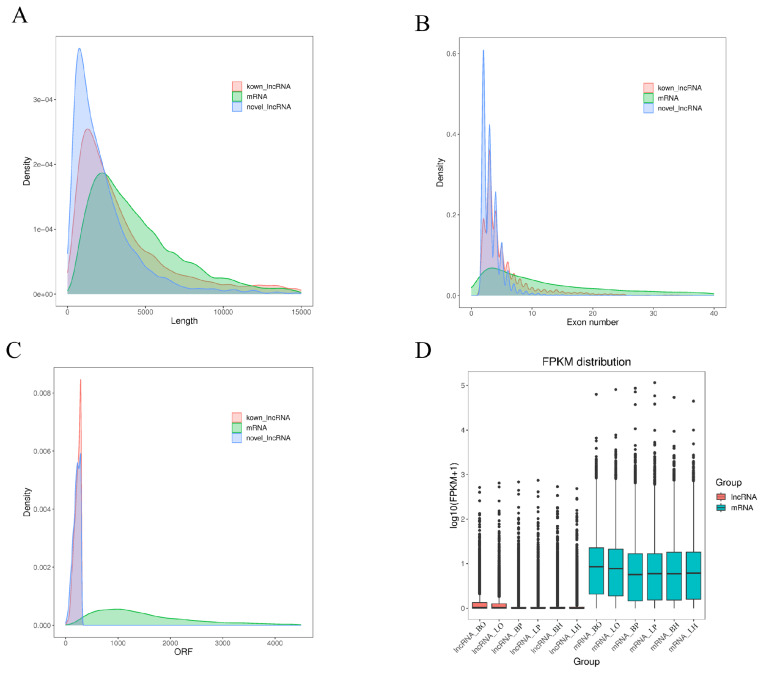
lncRNA genome characteristics. (A) The transcript lengths of lncRNA and mRNA. (B) The number of exons of lncRNA and mRNA. (C) The length of open reading frame of lncRNA and mRNA. (D) lncRNA and mRNA expression. lncRNA, long non-coding RNA; mRNA, messenger RNA.

The average length of the new lncRNA was 2,660 bp, much lower than the 5,163 bp of known lncRNA transcripts and 4,730 bp of mRNA transcripts (Figure 1A). The average number of exons of the new lncRNA was 3.4, and the average number of exons of known lncRNA was 5.4, much lower than the average number of mRNA exons (43.8) (Figure 1B). The ORF length of the new lncRNA and the known lncRNA is 307,484 and 704,358 amino acids, respectively, much lower than that of mRNA 9,351,376 amino acids (Figure 1C). Further, mRNA expression was much higher than that of lncRNA (Figure 1D). In summary, lncRNA has shorter transcript lengths, fewer exons, shorter ORF lengths, and lower expression levels than mRNA.

### Serum hormone level expression and biochemical index determination

**Figure 2:**
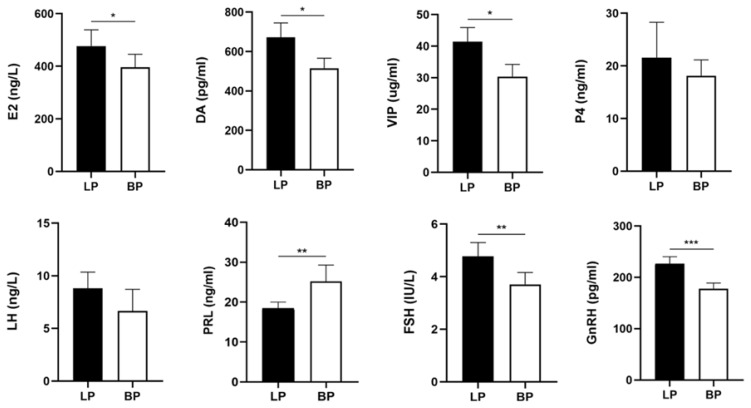
Serum reproductive hormones of Wanxi white geese during broodiness and laying period. * p<0.05, ** p<0.01, *** p<0.001. LP, laying period pituitary; BP, brooding period pituitary.

**Figure 3:**
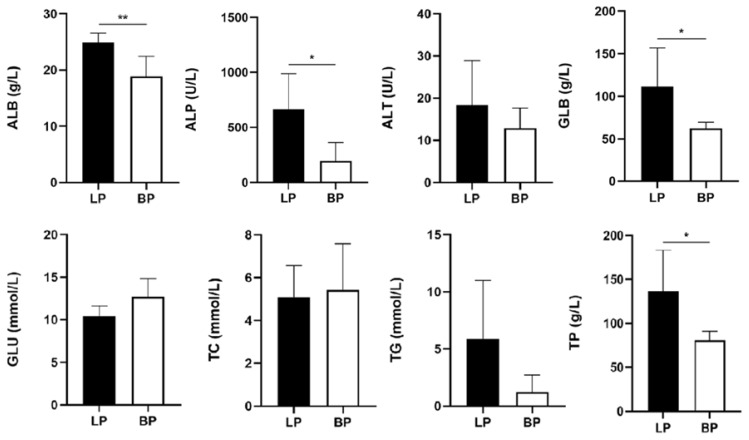
Serum biochemical indexes of Wanxi white geese during broodiness and laying period. * p<0.05, ** p<0.01. ALB, albumin; ALP, alkaline phosphatase; ALT, alanine aminotransferase; GLB, globulin; GLU, glucose; TC, total cholesterol; TG, triglycerides; TP, totalprotein; LP, laying period pituitary; BP, brooding period pituitary.

The serum hormones and biochemical indexes of laying period and broodiness period were measured to reflect the difference of serum hormones and serum biochemical expression between laying period and broodiness period. The levels of E2, DA and VIP in serum of WWG at laying stage were significantly higher than those at broodiness stage (Figure 2). The concentrations of P4 and LH in laying period were higher than those in broodiness period. The concentrations of FSH and GnRH in laying serum were significantly higher than those in brooding WWG. The PRL in the broodiness period was significantly higher than that in the laying period. In addition, compared with the brooding period, the levels of ALB, ALP, GLB and TP in the laying period were significantly higher than those in the nesting period; the concentration of ALT and TG in laying period was higher than that in brooding period. The concentrations of GLU and TC in the nesting period were lower than those in the laying period (Figure 3).

### Differentially expressed long non-coding RNA

**Figure 4:**
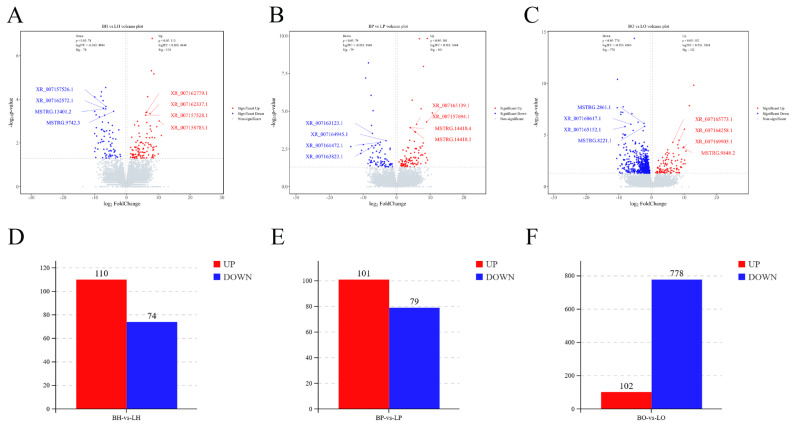
Volcano plots and histograms of differentially expressed lncRNA (|log2FC|>0.26, p<0.05). (A) and (D), (B) and (E), (C) and (F) represent BH vs LH, BP vs LP, BO vs LO. BH, brooding period hypothalamus; LH, laying period hypothalamus; BP, brooding period pituitary; LP, laying period pituitary; BO, brooding period ovary; LO, laying period ovary; lncRNA, long non-coding RNA.

**Figure 5:**
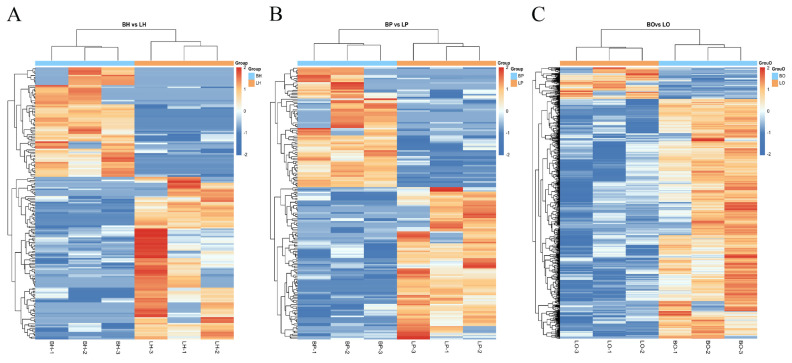
Differential gene expression heatmap. “Red” represents low relative expression level, and “blue” represents high relative expression level. Each column and row represent a sample and a gene, respectively. (A) BH and LH group. (B) BP and LP groups. (C) BO and LO group. BH, brooding period hypothalamus; LH, laying period hypothalamus; BP, brooding period pituitary; LP, laying period pituitary; BO, brooding period ovary; LO, laying period ovary.

Dynamic volcanogram and histogram reflected the differential expression of lncRNA between groups. The BH vs LH group had 184 DElncRNAs (110 up-regulated and 74 down-regulated) (Figures 4A, 4D). There were 180 DElncRNAs (101 up-regulated and 79 down-regulated) in the BP vs LP group (Figures 4B, 4E) and 880 DElncRNAs (102 up-regulated and 778 down-regulated) in the BO vs LO group (Figures 4C, 4F). Hierarchical clustering of the DEGs revealed that visually reflects the expression patterns of the genes in the samples and highlights the reproducibility and credibility of the data (Figure 5).

### Gene Ontology analysis of differentially expressed long non-coding RNA cis/trans target genes

**Figure 6:**
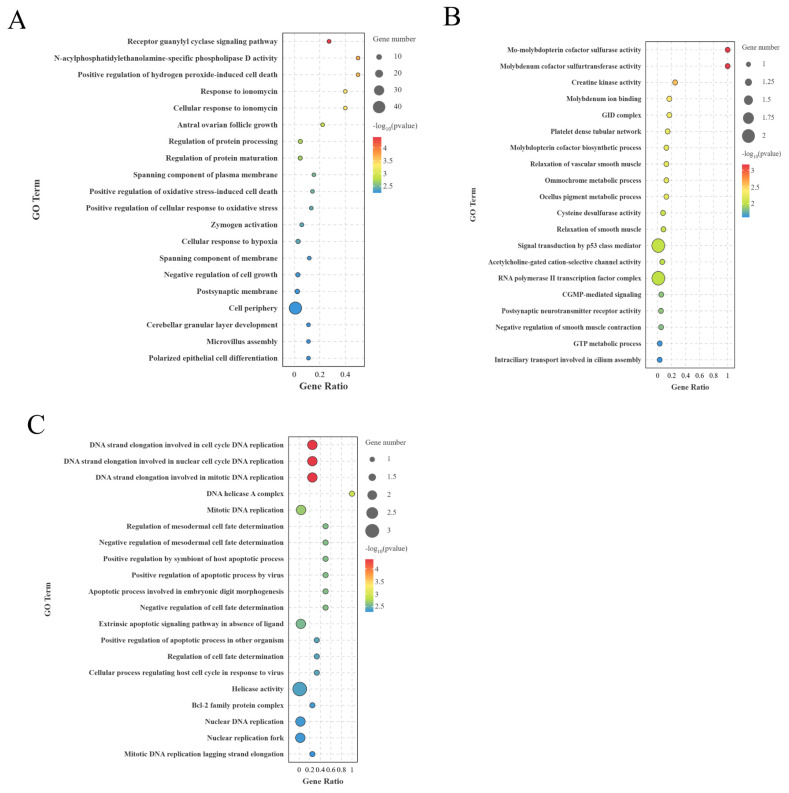
GO enrichment of lncRNA cis-target genes between different groups (top 20 GO entries). (A) BO and LO group. (B) BH and LH groups. (C) BP and LP group. GO, Gene Ontology; lncRNA, long non-coding RNA; BO, brooding period ovary; LO, laying period ovary; BH, brooding period hypothalamus; LH, laying period hypothalamus; BP, brooding period pituitary; LP, laying period pituitary.

The protein-coding genes within 100 kb of lncRNAs were considered as target genes for cis-regulation of DElncRNAS in the GO and KEGG enrichment analysis. BO and LO groups had 114 potential cis-regulatory target genes that significantly enriched in 595 entries (p<0.05). The entries mainly included ovarian antral follicle growth, postsynaptic membrane, and positive regulation of hydrogen peroxide-induced cell death (Figure 6A). Further, the BH vs LH group had 19 potential cis-regulatory target genes that significantly enriched 238 entries (p<0.05), mainly relaxation of vascular smooth muscle, neurotransmitter receptor activity after protrusion, and GTP metabolic process (Figure 6B). The BP vs LP had 21 potential cis-regulatory target genes that significantly enriched 115 entries (p<0.05), mainly cell cycle, DNA strand elongation involved in DNA replication, and DNA strand elongation involved in nuclear cell cycle DNA replication (Figure 6C).

**Figure 7:**
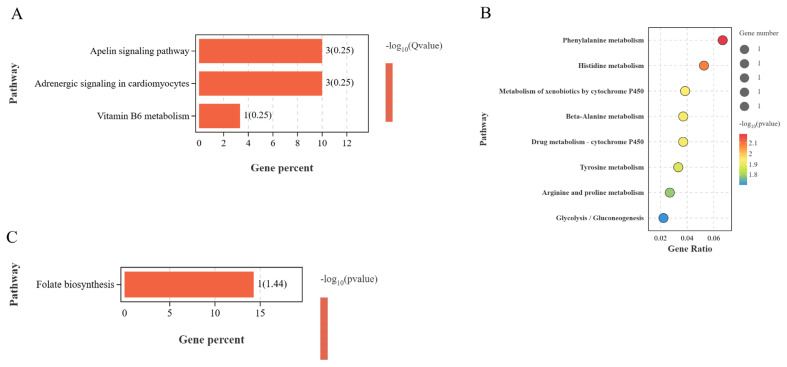
KEGG enrichment of lncRNA cis-target genes between different groups. (A) BO and LO group. (B) BH and LH group. (C) BP and LP group. KEGG, Kyoto Encyclopedia of Genes and Genomes; lncRNA, long non-coding RNA; BO, brooding period ovary; LO, laying period ovary; BH, brooding period hypothalamus; LH, laying period hypothalamus; BP, brooding period pituitary; LP, laying period pituitary.

KEGG enrichment analysis showed that most of the pathways significantly enriched by these cis-regulated lncRNAs target genes were related to follicular development and broodiness. For example, the BO vs LO group significantly enriched three pathways (p<0.05), mainly the apelin signaling pathway, adrenergic signaling in cardiomyocytes, and vitamin B6 metabolism (Figure 7A). The BP vs LP group had eight significantly enriched pathways (p<0.05), mainly phenylalanine metabolism, metabolism of xenobiotics by cytochrome P450, and tyrosine metabolism (Figure 7B). Folate biosynthesis was the only significantly enriched pathway in the BH vs LH group (Figure 7C). Therefore, lncRNAs may affect ovarian follicular development in WWG during laying and broodiness via cis-regulation of adjacent protein-coding genes.

**Figure 8:**
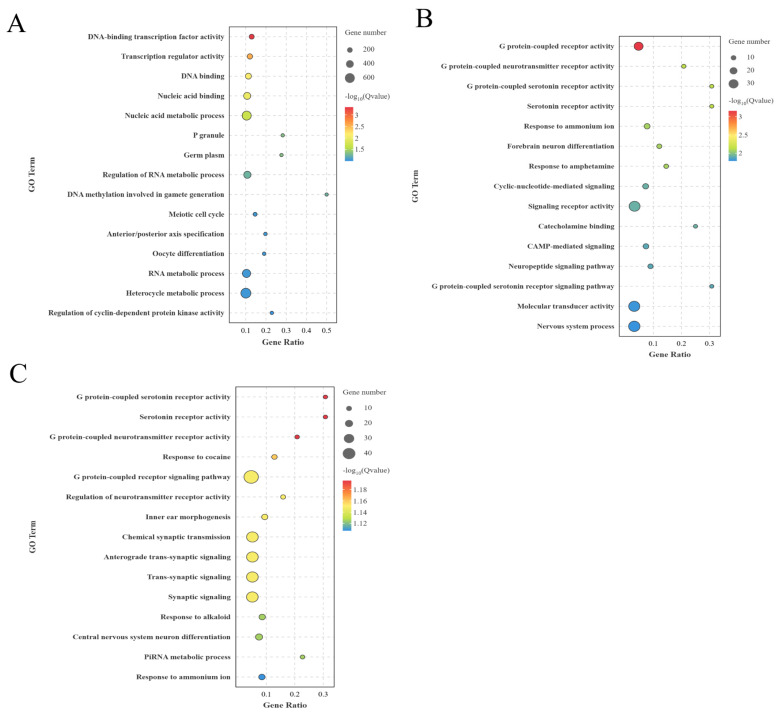
GO enrichment of lncRNA trans-target genes between different groups (top 15 GO entries). (A) BO and LO group. (B) BH and LH groups. (C) BP and LP group. GO, Gene Ontology; lncRNA, long non-coding RNA; BO, brooding period ovary; LO, laying period ovary; BH, brooding period hypothalamus; LH, laying period hypothalamus; BP, brooding period pituitary; LP, laying period pituitary.

The BO vs LO group had DE 1,530 potential lncRNAs trans-regulated target genes, which significantly enriched 686 entries (p<0.05), mainly oocyte differentiation, meiotic cell cycle, and regulation of biosynthesis process (Figure 8A). Further, the BH vs LH group had 312 potential target genes which significantly enriched 815 entries (p<0.05), mainly the neuropeptide signaling pathway, intercellular signal transduction, and cAMP-mediated signal transduction (Figure 8B). BP vs LP had 459 potential target genes that significantly enriched 730 entries (p<0.05), mainly the G protein-coupled neurotransmitter receptor activity, oocyte nucleolus formation, and neuronal differentiation of the central nervous system (Figure 8C).

**Figure 9:**
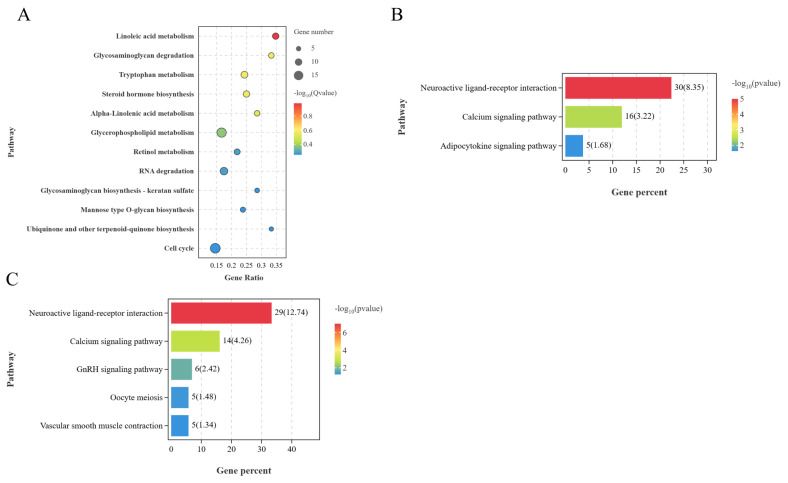
KEGG enrichment analysis of lncRNA trans-target genes between different groups. (A) BO and LO group. (B) BH and LH group. (C) BP and LP group. KEGG, Kyoto Encyclopedia of Genes and Genomes; lncRNA, long non-coding RNA; BO, brooding period ovary; LO, laying period ovary; BH, brooding period hypothalamus; LH, laying period hypothalamus; BP, brooding period pituitary; LP, laying period pituitary.

KEGG enrichment analysis of the identified lncRNA trans-target genes revealed 12 significantly enriched pathways in the BO vs LO group (p<0.05), mainly steroid hormone biosynthesis, glycerophospholipid metabolism, and retinol metabolism (Figure 9A). The genes from the BP vs LP group significantly enriched three pathways (p<0.05), mainly neuroactive ligand-receptor interaction, calcium signaling, and adipocytokine signaling (Figure 9B). There were five significantly enriched pathways in the BH vs LH group (p<0.05), mainly GnRH signaling, calcium signaling, and oocyte meiosis (Figure 9C). Therefore, lncRNAs may affect ovarian follicular development of laying and brooding WWG through the trans-regulation of non-adjacent protein-coding genes.

### Validation using real-time fluorescence quantitative reverse transcription polymerase chain reaction

**Figure 10:**
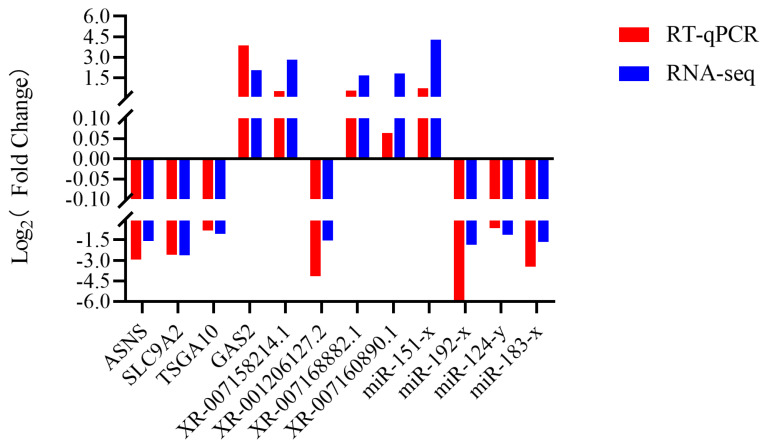
RT-qPCR validation using DEGs, DElncRNAs, and DEMs. Red and blue represent RT-qPCR results, respectively. RT-qPCR, reverse transcription quantitative polymerase chain reaction; DEGs, differentially expressed genes; DElncRNA, differentially expressed long non-coding RNA; DEMs, differentially expressed miRNA.

Randomly selected 4 DEGs, 4 DEMs, and 4 lncRNAs were used for reverse transcription quantitative polymerase chain reaction (RT-qPCR) validation of sequencing results. The results showed that DEG, DEM, and DElncRNA expression trends in different groups of sample tissues were consistent with the transcriptome sequencing, indicating that the RNA-seq data is reliable (Figure 10).

### The long non-coding RNA-microRNA-messenger RNA interaction network

**Figure 11:**
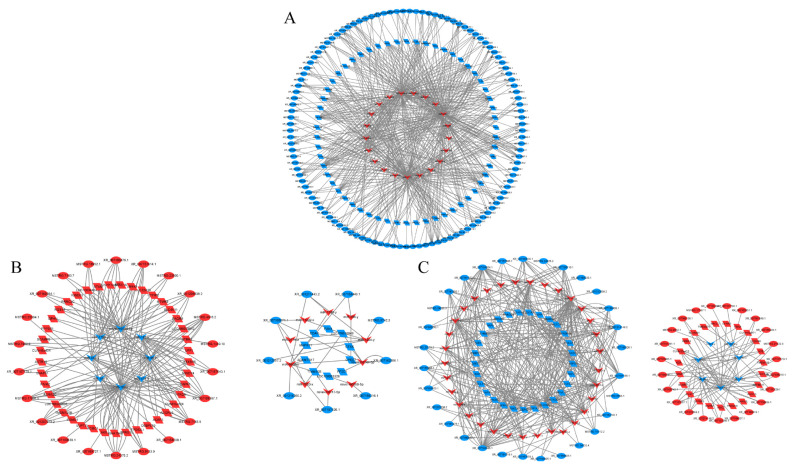
Interaction network of lncRNA-miRNA-mRNA between different groups. (A) BO and LO groups. (B) BH and LH groups. (C) BP and LP group. Blue and red indicate down-regulation and up-regulation, respectively. lncRNA, long non-coding RNA; miRNA, microRNA; mRNA, messenger RNA; BO, brooding period ovary; LO, laying period ovary; BH, brooding period hypothalamus; LH, laying period hypothalamus; BP, brooding period pituitary; LP, laying period pituitary.

In order to elucidate the biological mechanism of lncRNAs in ovarian follicular development, we used bioinformatics tools to construct a regulatory network containing these lncRNAs, miRNAs, and their potential target genes. The BO vs LO group has 201 nodes and 1,172 edges, including 116 DElncRNAs (3 up-regulated and 113 down-regulated), 62 DEGs (3 up-regulated and 59 down-regulated), and 23 DEMs (21 up-regulated and 2 down-regulated) (Figure 11A). The central nodes include XR_007167835.1, XR_007163297.1, KNDC1, RIMS4, miR-277-z, and miR-339-x. The BH vs LH group has 72 nodes and 312 edges, including 23 DElncRNAs (17 up-regulated and 6 down-regulated), 50 DEGs (38 up-regulated and12 down-regulated), and 10 DEMs (4 up-regulated and 6 down-regulated) (Figure 11B) The central nodes include MSTRG.7163.5, XR_007168367.1, PAQR9, DAPK1, miR-182-x, and miR-151-x. Further, the BP vs LP group has 145 nodes and 940 edges, including 53 DElncRNAs (23 up-regulated and 30 down-regulated), 40 DEGs (32 up-regulated and 8 down-regulated), and 40 DEMs (32 up-regulated and 8 down-regulated) (Figure 11C). The central nodes include XR-007164945.1, XR_007161186.1, PLPPR5, GAD1, miR-144-y, and miR-276-y.

**Figure 12:**
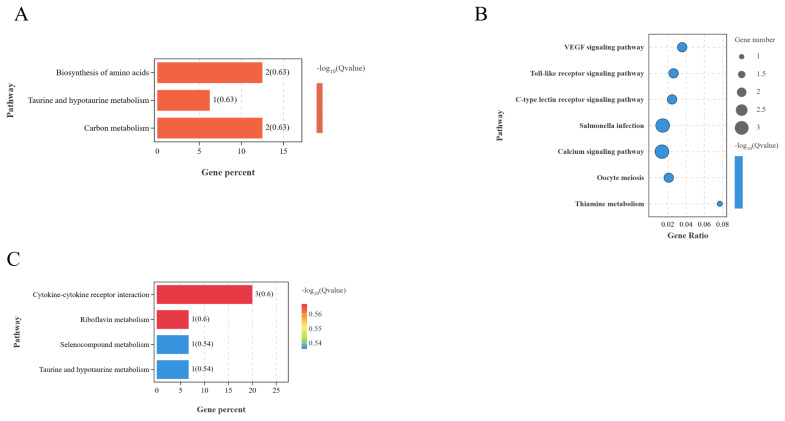
KEGG diagram of interaction networks between different groups. (A) BO and LO groups. (B) BH and LH groups. (C) BP and LP group. KEGG, Kyoto Encyclopedia of Genes and Genomes; BO, brooding period ovary; LO, laying period ovary; BH, brooding period hypothalamus; LH, laying period hypothalamus; BP, brooding period pituitary; LP, laying period pituitary.

The KEGG functional enrichment using mRNAs involved in the ceRNA network of the BO vs LO significantly enriched biosynthesis of amino acids, taurine and hypotaurine metabolism, and carbon metabolism (p<0.05) (Figure 12A). Those of BH vs LH significantly enriched oocyte meiosis, toll-like receptor signaling, VEGF signaling, and thiamine metabolism pathways (p<0.05) (Figure 12B). Finally, those of BP vs LP significantly enriched cytokine-cytokine receptor interaction, sulfur compound metabolism, and riboflavin metabolism pathways (p<0.05) (Figure 12C).

### Dual-luciferase reporter assay system

**Figure 13:**
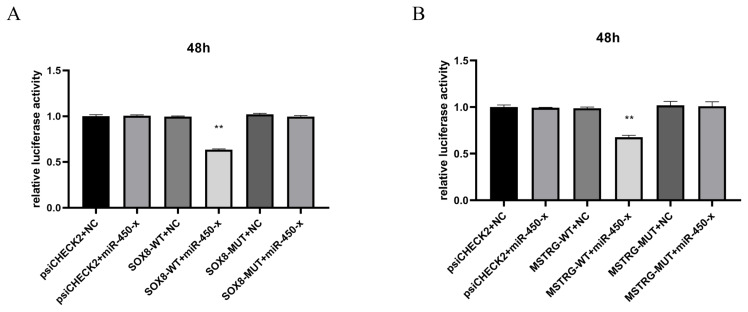
Luciferase reporter gene assay. (A) A dual luciferase reporter assay detected the SOX8 and miR-450-x interaction. (B) The interaction between MSTRG.1166.1 and miR-450-x was detected by dual luciferase reporter assay. ** p<0.01.

MSTRG.1166.1 and SOX8 were down-regulated in the BO vs LO group, and miR-450-x was up-regulated in the BO vs LO group. RNAhybrid, miRanda, and TargetScan software predicted that MSTRG.1166.1 and SOX8 targeted miR-450-x. Therefore, a mutant vector was constructed to verify the specific binding site. The dual luciferase activity ratio of the co-transfection of miR-450-x and SOX8-3′UTR-WT was significantly down-regulated compared to the control group (p<0.05). There was no significant difference between the ratio of miR-450-x and SOX8 mRNA 3′UTR-MUT co-transfection dual luciferase activity and the control (p>0.05). The ratio of miR-450-x and MSTRG.1166.1-WT co-transfected dual luciferase activity was significantly low compared to the control group (p<0.05). Finally, the ratio of miR-450-x and MSTRG.1166.1-MUT co-transfection dual luciferase activity was not significantly different from the control group (p>0.05). Therefore, MSTRG.1166.1 and SOX8 directly target miR-450-x (Figure 13).

## DISCUSSION

The ovary is among the most important reproductive organs in poultry, containing many follicles at different developmental stages. At different follicular developmental stages, various reproductive endocrine hormones, paracrine and autocrine regulatory factors, and the regulatory activities of the HPG axis strictly control the ovary. This study showed that the serum hormone (E2, DA, and VIP) levels in the serum of laying WWG were significantly higher than those in the brooding period. Furthermore, the concentrations of FSH and GnRH in laying birds were significantly higher than those in the broodiness period. The PRL in the broodiness period was significantly higher than that in the laying period, and the concentrations of P4 and LH in the laying period were higher than those in the broodiness period. This study found that increasing PRL may be a key factor in inducing poultry broodiness. Therefore, the hormones released from the hypothalamus and pituitary may affect ovarian follicular development in WWG, leading to egg production and brooding behavior. Our previous study revealed significant differences in ovarian histological morphology between laying and brooding WWG geese [15]. In summary, the physiological changes, reproductive performance, and ovarian tissue structure of WWG during the laying and brooding periods may cause the occurrence of brooding in WWG. Therefore, the HPG axis is essential for ovarian development and laying performance in WWG at different laying stages, and it is regulated by various genes, transcription factors, and lncRNAs. Nevertheless, the DElncRNAs and key pathways that mediate egg-laying within the HPG axis remain unclear.

In this study, the hypothalamus, pituitary, and ovarian tissues of laying and broodiness WWG were analyzed for differential lncRNA expression. The potential functions of DElncRNAs were analyzed by co-localization and co-expression of related genes. The ceRNA regulatory network of the predicted DElncRNA-bound miRNAs displayed the potential functions of DElncRNAs. Furthermore, GO and KEGG enrichment of the DElncRNA cis-target and trans-target genes revealed three groups of DEGs that significantly enriched biological processes such as biological process regulation, catalytic activity, and cell composition (p<0.05). KEGG showed that the three groups of differentially expressed cis-target genes and trans-target genes significantly enriched multiple signaling pathways related to reproductive performance (p<0.05). The pathways include apelin signaling, steroid hormone biosynthesis, neuroactive ligand-receptor interaction, and calcium signaling pathways. The apelin signaling pathway regulates reproductive hormone secretion by affecting the HPG axis and the egg production cycle [16]. Steroid hormones such as E2 and transforming growth factor β1 (TGF-β1) of the steroid hormone biosynthesis pathway play an important regulatory role in avian ovarian development. Estrogen can promote follicle activation and development, increasing the number of growing follicles, thus promoting follicular cell proliferation and the activation and development of primordial follicles. The levels of P4 and E2 vary in follicles at different developmental stages and these changes reflect the different stages of follicular development, playing an important role in regulating follicle growth and maturation. This explanation is consistent with the changes in the contents of P4 and E2 in the blood of WWG during the laying and broodiness periods (Figure 2). The TGF-β1 belongs to the TGF-β subfamily, which can stimulate the expression of the FSH receptor and amplify the FSH-induced aromatase activity [17,18]. The neuroactive ligand-receptor interaction pathway and receptor protein tyrosine kinase may play a key role in the regulation of ovarian function and egg production [19]. The calcium signaling pathway regulates oocyte maturation, and the balance of calcium ions has an important effect on oocyte maturation and embryonic development [20]. Within the above-mentioned signaling pathways, three target genes of DElncRNAs associated with broodiness and egg-laying performance were identified: CGA, CRH, and DRD1. Among these, the CGA gene is closely linked to FSH and LH secretion and promotes follicular maturation. CRH mainly regulates the secretory activity of the pituitary and hypothalamus and participates in follicular development. DRD1 indirectly modulates the avian reproductive system and broodiness by regulating prolactin (PRL) secretion via dopaminergic signaling. Therefore, we hypothesize that the target genes of these DElncRNAs collectively participate in the regulation of broodiness in WWG geese via the hypothalamic–pituitary–gonadal (HPG) axis.

In this study, the ceRNA interaction network was constructed by combining DEGs and DEMs found in the previous study of our team with DElncRNAs found in this study for identifying the candidate lncRNAs that control the laying performance and broodiness behavior of WWG. miR-277-z had the most nodes in the ceRNA network of BO vs LO, with *RAB3B* and *PRPS2* as the predicted target genes being negatively correlated with miR-277-z expression. Previous studies have shown, elevated miR-277-3p suppresses dopa decarboxylase (DDC) expression at both the mRNA and protein levels, thereby affecting reproductive function [21]. *RAB3B* exists in the ovarian tissue and is co-localized with oxytocin in the same corpus luteum staining granules during the luteal phase of the ovine estrous cycle. Therefore, the *RAB3B* protein is directly or indirectly related to the hormone secretion pathway of the corpus luteum. Additionally, *RAB3B* is involved in GnRH-induced pituitary gonadotropin release, which affects FSH and LH synthesis, consistent with the significantly lower expression of *RAB3B* in the ovarian tissue during the broodiness than the laying period [22]. The concentrations of FSH, GnRH, and LH in the blood of WWG during the laying period were significantly higher than those during the broodiness period (Figure 2). In the ceRNA network, XR_007167835.1 has more nodes, probably targets miR-277-z, and is negatively correlated with miR-277-z. In this study, XR_ 007167835.1 and *RAB3B* expression in the ovarian tissues were significantly higher during the laying than during the broodiness period. The expression of miR-277-z in the ovarian tissue was significantly higher in broodiness than in the laying period. Therefore, XR _ 007167835.1-miR-277-z-*RAB3B* may affect follicle development in WWG by affecting LH and FSH synthesis. Moreover, the ceRNA network for miR-339 had more nodes. miR-339 can directly bind to the PII region of the CYP19A1 promoter and activates CYP19A1 transcription by changing histone modifications, thereby enhancing E2 release [23]. *TRAF4* is a target gene of miR-339-x, and TRAF4 affects apoptosis by interacting with the dimer neurotrophin receptor p75 (NTR) [24]. MSTRG.10196.1/XR_001206277.2 targeted miR-339-x and was negatively regulated by miR-339-x. In this study, MSTRG.10196.1/XR_001206277.2 and *TRAF4* expression were significantly higher in ovarian tissues during the broodiness period than during the laying period. Further, miR-339-x expression was significantly higher in ovarian tissue during the laying than the brooding period, and the E2 concentration in serum was significantly higher during the laying than the brooding period (Figure 2). Therefore, MSTRG.10196.1 /XR _001206277.2-miR-339-x-*TRAF4* may affect the broodiness behavior and laying performance of WWG by affecting E2 synthesis. MiR-450-x also has more nodes in the ceRNA network diagram. Overexpressing miR-450-5p can increase granulosa cell proliferation [25]. Furthermore, *SOX8*, a predicted target gene of miR-450-x, may induce ovarian dysfunction and is associated with ovarian cell apoptosis [26]. There is a negative regulation between *SOX8* and miR-450-x. Therefore, MSTRG.1166.1 affects *SOX8* expression by targeting miR-450-x. Previous studies have shown, miR-450-5p is involved in follicular maturation in goats; its high expression promotes the proliferation of follicular granulosa cells [25]. Additionally, a dual luciferase reporter gene assay showed that the miR-450-x target site in MSTRG.1166.1 sequence, *SOX8* mRNA3′-UTR. MSTRG.1166.1, and *SOX8* were low expression in ovarian tissue, and miR-450-x was highly expressed in ovarian tissue. Therefore, MSTRG.1166.1-miR-450-x-*SOX8* may regulate ovarian development in WWG by affecting granulosa cell proliferation and apoptosis.

In the BH vs LH group, miR-182-x had the most nodes in the ceRNA network, while miR-182 inhibited cell proliferation and promoted apoptosis [27]. *CSMD1* is a predicted miR-182-x target gene and is negatively correlated with miR-182-x expression. Furthermore, *CSMD1* is highly expressed in the oocytes of developing follicles. A *CSMD1* loss function may lead to premature macrophage invasion of the developing follicles, resulting in excessive oocyte atresia and reduced ovulation [28]. In the ceRNA network, MSTRG.7163.5 has more nodes, probably targets miR-182-x, and is negatively correlated with miR-182-x. Studies have shown, Studies have shown that miR-182-5p is expressed in the duck hypothalamus and primarily influences lipid metabolism, proteolysis, endocrine signaling, and neurotransmission [29]. In this study, the expression of MSTRG.7163.5 and *CSMD1* were significantly lower in the hypothalamus during the broodiness than the laying period. Besides, miR-182-x expression was significantly higher in the hypothalamus during the laying than in the broodiness period. Therefore, MSTRG.7163.5-miR-182-x-*CSMD1* may affect the egg-laying performance of the WWG by affecting oocyte development. The node ranking of miR-151-y in the ceRNA network was second. High levels of miR-151-3p in the follicular fluid can promote oocyte maturation and embryonic development [30]. *PAQR9* is the target gene of miR-151-y and a member of the progesterone receptor subfamily in the progesterone adiponectin receptor family (mPRs), an important receptor family on the cell membrane. This mPRs family has a typical G protein-coupled receptor transmembrane structure. mPRa mRNA and protein are located in the main reproductive organs of zebrafish ovary, testis, and pituitary gland, where mPRa mediates progesterone function and signal transduction in various animal models and cell types [31]. MSTRG.7163.5 targets miR-151-y and is negatively regulated by miR-151-y. In this study, E2 and P4 concentrations were higher in the blood of WWG during the laying than the broodiness period (Figure 2). The expression of MSTRG.7163.5 and *PAQR9* were significantly higher in the hypothalamus during the broodiness than laying period. Furthermore, miR-151-y expression was significantly higher in the hypothalamus during laying than broodiness. Therefore, MSTRG.7163.5-miR-151-y-*PAQR9* may affect follicular development in WWG by affecting progesterone synthesis. MiR-96 is a key mediator of ovulatory LH surge [32]. In the ceRNA network, *DAPK1* is a predicted target gene of miR-96-x, and *DAPK1* inhibits ovarian granulosa cell apoptosis [33]. In this study, the concentration of LH in the blood of WWG was higher during the laying than during the broodiness period. Furthermore, MSTRG.4615.2 and *DAPK1* expression was significantly higher in the hypothalamus during the laying than in the broodiness period. miR-96-x expression was significantly higher in the hypothalamus during the broodiness than during the laying period. Therefore, MSTRG.4615.2-miR-96-x-*DAPK1* may affect follicle development in WWG by affecting LH synthesis.

In the ceRNA network of BP vs LP, miR-144-y had the most regulatory points in the network. Overexpressing miR-144-y reduced granulosa cell apoptosis by inhibiting the downstream p38 MAPK signaling pathway [34]. *TFPI* is a predicted target of miR-144-y and negatively correlates with miR-144-y expression. *TFPI* is related to steroid synthesis and follicular growth and development [35]. In the ceRNA network, XR_007164924.1 targets miR-144-y and has a negative regulatory relationship with miR-144-y. In this study, XR_ 007164924.1 and *TFPI* expression were significantly higher in the pituitary tissues during the broodiness than during the laying period. However, miR-144-y expression was significantly higher in the pituitary tissue during the laying than in the brooding period. XR_007164924.1-miR-144-y-*TFPI* may affect follicular development in WWG by affecting steroid hormone synthesis. Overexpressing miR-205 promotes granulosa cell apoptosis and reduces estradiol synthesis [36]. *THRB*, the predicted target of miR-205-x, is negatively correlated with miR-205-x expression. *THRB* encodes the thyroid hormone receptorβ, and its abnormal expression is related to the regulation of ovarian development, follicular atresia, steroidogenesis, and egg production arrest in chickens [37]. In this study, XR_007161186.1 and *THRB* expression were low during laying, and miR-205-x was highly expressed during the laying period. XR_007161186.1 targeted miR-205-x and had a negative regulatory relationship with miR-205-x. Therefore, XR_ 007161186.1-miR-205-x-*THRB* may affect the proliferation and apoptosis of the granulosa cells of WWG by affecting steroid hormone synthesis. MiR-9-y may affect the proliferation and apoptosis of ovarian granulosa cells [38]. *FBN3*, a microfibrin in the extracellular matrix, is highly expressed in early ovarian development [39]. The ceRNA network revealed that *FBN3* was the target of miR-9-y, and there was a negative regulation between *FBN3* and miR-9-y. At the same time, MSTRG.9442.1 targets miR-9-y and has a positive regulation with miR-9-y. This study showed that MSTRG.9442.1 and *FBN3* were highly expressed during laying, and miR-9-y expression was low during the laying period. MSTRG.9442.1-miR-9-y-*FBN3* can potentially regulate the apoptosis and proliferation of granulosa cells in WWG.

## CONCLUSION

This study sequenced and analyzed the lncRNAs in the hypothalamus, pituitary, and ovarian tissues of brooding and laying WWG. DElncRNAs are related to laying performance and broodiness. Functional enrichment showed that most of the DElncRNAs target genes significantly enriched apelin signaling, steroid hormone biosynthesis, neuroactive ligand-receptor interaction, calcium signaling pathway, and other pathways. Additionally, the ceRNA regulatory network involved in the ovarian development of WWG was constructed. The preliminary predicted MSTRG.1166.1-miR-450-x-*SOX8*, MSTRG.7163.5-miR-182-x- *CSMD1*, XR_007167835.1-miR-277-z-*RAB3B*, MSTRG.10196.1/XR_001206277.2-miR-339-x-*TRAF4*, MSTRG.7163.5-miR-151-y-*PAQR9*, MSTRG.4615.2-miR-96-x-*DAPK1*, XR_007 164924.1-miR-144-y-*TF-PI*, XR_007161186.1-miR-205-x-*THRB*, and MSTRG.9442.1-miR-9-y-*FBN3* may play an important role in the ovarian development of WWG. These results provide an important reference for revealing the regulatory mechanism of reproductive performance in WWG. Further, it lays a foundation for further understanding the genetic mechanism of laying performance and broodiness behavior of WWG.

## Data Availability

The original sequencing data for the hypothalamus, pituitary, and ovary of Wanxi White geese have been uploaded to the NCBI database under BioProject ID: PRJNA1169106.

## SUPPLEMENTARY MATERIAL

**Supplement 1**. The primers of the mRNAs, lncRNAs and miRNAs and for the RT-qPCR.
