# Supplementary material for: Differential expression of long non-coding RNA in the hypothalamus-pituitary-gonadal axis of Wanxi white geese during laying and broodiness periods
Source: Anim Biosci. 2025 Oct 22;39(3):250348. doi: 10.5713/ab.25.0348 (PMC12963733; doi:10.5713/ab.25.0348)
Supplement: Supplementary file 1 [file ab-25-0348-Supplementary-1.pdf]

**Supplement 1.** The primers of the mRNAs、lncRNAs and miRNAs and for the RT-qPCR

| Gene Name | Primer sequences（5’-3’） | Amplicon size（bp） | T（℃） |
|---|---|---|---|
| *ASNS* | F： ctgcagaagagagtgagagg<br>R： gggactctcagttcaagacc | 100 | 57 |
| *SLC9A2* | F： gaagacttctcatccgggag<br>R： ctgatcattccactctcggc | 106 | 58 |
| *TSGA10* | F：AACAAAATGCCGTCAAGCAG<br>R：ATTAGTGCCAGTCTAACAGAGC | 54 | 60 |
| *GAS2* | F：TTTGCACGAGACAACACAGC<br>R：AACAGGCAGGTATCATCCAC | 71 | 60 |
| *XR_007158214.1* | F: CCTGTTGATCGAGTGTCTCAG<br>R: CCAGATTCGACTCTCAGTCAC | 117 | 58 |
| *XR_001206127.2* | F: CTTCTATCTTGAGCTCGTCCC<br>R: GAGAGCAGACTATGGCAGAAG | 113 | 58 |
| *XR_007168882.1* | F: GAACCAATGGTAGAGGGTGAG<br>R: CAGAATATGCGTCCCTGAGAG | 100 | 58 |
| *XR_007160890.1* | F: CTTGAAGCCAGAGATCCTCAC<br>R: GCCACATCAAGGGTATCAGAG | 98 | 58 |
| *GAPDH* | F：AGCCATCAATGATCCCTT<br>R：ATTCTCAGCCTTGACTGTG | 100 | 60 |
| miR-151-x | RT:<br>GTCGTATCCAGTGCAGGGTCCGAGGT<br>ATTCGCACTGGATACGACACTAGA<br>F: CGCGTCGAGGAGCTCACAG | | 60 |
| miR-192-x | RT：<br>GTCGTATCCAGTGCAGGGTCCGAGGT<br>ATTCGCACTGGATACGACGGCTGT<br>F：GCGCGCTGACCTATGAATTG | | 60 |
| miR-124-y | RT：<br>GTCGTATCCAGTGCAGGGTCCGAGG<br>TATTCGCACTGGATACGACGGCATT<br>F：GCGTAAGGCACGCGGTG | | 60 |
| miR-183-x | RT：<br>GTCGTATCCAGTGCAGGGTCCGAGG<br>TATTCGCACTGGATACGACAGTGAA<br>F：CGCGTATGGCACTGGTAGAA | | 60 |
| U6 | RT：AACGCTTCACGAATTTGCGT<br>F：CTCGCTTCGGCAGCACA<br>R：AACGCTTCACGAATTTGCGT | 68 | 60 |
